# Identification of New Players in Hepatocarcinogenesis: Limits and Opportunities of Using Tissue Microarray (TMA)

**DOI:** 10.3390/microarrays3020091

**Published:** 2014-04-15

**Authors:** Luca Quagliata, Manuel Schlageter, Cristina Quintavalle, Luigi Tornillo, Luigi M. Terracciano

**Affiliations:** Molecular Pathology Division, Institute of Pathology, University Hospital of Basel, CH-4031 Basel, Switzerland; E-Mails: Manuel.Schlageter@usb.ch (M.S.); cristina.quintavalle@usb.ch (C.Q.); luigi.tornillo@usb.ch (L.T.); lterracciano@uhbs.ch (L.M.T.)

**Keywords:** TMA, liver, Hepatocellular carcinoma, IHC

## Abstract

Liver tumours are among the leading causes of cancer-related death worldwide and hepatocellular carcinoma (HCC) accounts for the vast majority of liver tumours. When detected at an early stage of disease, patients might still be eligible for surgical-based curative treatments. However, currently only small portion of HCC affected patients are diagnosed at an early stage. For late stage HCC no treatment option exists beside the multi-tyrosine kinase inhibitor Sorafenib. Thus new molecular targets and treatment options for HCC are urgently needed. Nevertheless, despite some improvements in diagnosis and patient management, the biology of liver tumour remains inadequately understood, mainly because these tumours have shown to harbour a highly complex genomic landscape. In addition, one major obstacle delaying the identification of new molecular targets in biomedical research is the necessity to validate them using a large collection of tissue specimens. Tissue microarray (TMA) technology allows the prompt molecular profiling of multiple tissue specimens and is therefore ideal to analyze presumptive candidate biomarkers in a fast an effective manner. The use of TMA has substantial benefits over standard techniques and represents a significant advancement in molecular pathology. For example, TMA technology reduces laboratory work, offers a high level of experimental uniformity and provides a judicious use of precious tissue. On the other hand, one potential limitation of using TMA is that the small cores sampled may not be representative of whole tumors. This issue is very critical in particularly heterogeneous cancers such as HCC. For liver focused studies, it is ideal to evaluate the staining patters of a determined marker over the structure of an entire acinus and to define staining in as many as possible anatomical regions. In this review we analyze the limits and opportunities offered by the usage of TMA technology in HCC research. In summary, TMA has revolutionized the histopathological analysis and will be of great help to further advance the knowledge in the field of hepatocarcinogenesis research.

## 1. Introduction: HCC an Overview

Liver tumours are among the leading causes of cancer-related death worldwide and the principal cause of mortality among cirrhotic patients [1]. In contrast to other tumour entities, mortality from liver cancer has considerably increased over the past decades [2]. In addition, epidemiologic data about the prevalence of chronic hepatitis indicates that the medical and economic burden of liver cancers will still drastically increase in the next 15 years [2]. Hepatocellular carcinoma (HCC) accounts for 80% of all liver tumours, with the others being either cholangiocarcinoma (CC) or mixed forms. HCC mostly arises in patients suffering from a cirrhotic liver [3], with more than half of new cases (mainly in Eastern countries) being associated to chronic infection of either hepatitis B (HBV) or C (HCV) virus [4]. Besides established risk factors, such as male gender, older age, high levels of bilirubin, altered liver enzymes status, increased portal hypertension, ethnicity and viral genotypes [4,5,6], little is known about the mechanisms that favor HCC development and progression [6]. Notably, it is not clear why a subgroup of patients with cirrhosis will eventually develop HCC, whereas others do not. When detected at an early stage of disease, patients might be still eligible for surgical-based curative treatments. However, currently only 30% to 40% of HCC affected patients are diagnosed at an early stage and can undergo resection, local ablation or transplantation [6]. Finally, even complete tumor removal does not certainly offer a sheltered curative solution, as the underlying liver disease (mostly cirrhosis) will still persist in the remaining liver. In fact, about 80% of HCC patients experience recurrence after surgical resection [7,8,9]. Furthermore, if HCC is detected at an intermediate or advanced stage, no treatment option exists beside Sorafenib [10]. Sorafenib is multi-tyrosine kinase inhibitor that effectively blocks several receptors activity such as VEGFR (Vascular Endothelial Growth Factor Receptor), PDGFR (Platelet Derived Growth Factor Receptor) and the RAF serine/threonine kinases along the RAF/MEK/ERK pathway [10]. However, Sorafenib has shown a consistent but limited survival benefit in HCC (10 to 12 weeks increased survival) accompanied by a number of moderate to severe side effects [3,10]. Concerning HCC diagnosis, nowadays, histopathological assessment of specimens still remains the most effective and accurate option [4]. Such an approach is clearly dependent on the expertise of the pathologist revising the case and therefore suffers of inter-observer variability. While late stage HCC is usually a straightforward Haematoxylin & Eosin (H&E) diagnosis, early stage HCC is more problematic and often demands the evaluation of additional histological features, for example an assessment of the reticulin framework. Therefore, the identification of new molecular markers to specifically identify early HCC might result in an increased diagnostic output. Despite some improvements in diagnosis and patient management, the biology of liver tumour remains inadequately understood, mainly because these tumours have shown to harbour a highly complex genomic landscape [6]. Among the best-described pathways driving HCC, there are Wnt/β-catenin, MAPK, p14ARF/p53, p16INK4A/Rb, transforming growth factor-β (*TGF-β*) and PTEN/Akt [11]. Moreover, using comparative genomic hybridization (CGH) technology, HCC has shown frequent DNA copy number gains at chromosomes 1q, 8q and losses at 1p, 4q, 8p, 13q, 16q and 17q [12,13,14,15]. An accurate molecular classification should highlight drug-targets, such as growth factor receptors or kinases, thus allowing personalized targeted therapies. So far, several studies have attempted to establish a comprehensive HCC molecular classification, mostly based on gene expression profile [16,17,18]. However till now, none of such classifications have been validated in the clinical practice [19], mainly because of the many discrepancies between the presented models [20,21]. Thus the identification of specific molecular markers able to classify the different HCC subgroups and possibly identify early stage of disease onset is urgently needed.

## 2. TMA Methodology: A Historical Overview and Basic Concepts

Back in 1986, original work from Hector Battifora described a ‘sausage’ block method to prepare multi tissue sections that is considered the prototype of nowadays well-known tissue microarray (TMA) [22]. Battifora’s innovative approach however suffered of several limitations, as for example the inability to identify individual tissue rods, that were subsequently addressed by Kononen *et al.* [23] who shaped the TMA as we know it now. Kononen and colleagues introduced a novel sampling method to produce tissues of regular shape and defined size, making them more suitable to be densely and precisely arrayed. This TMA technology allows the prompt molecular profiling of multiple tissue specimens and is therefore ideal to analyze presumptive candidate biomarkers in a fast and effective manner [24,25]. The constant expanding knowledge produced by recent research in molecular biology has identified a plethora of novel presumptive biomarkers, which might have major diagnostic, prognostic and therapeutic significance [26]. However, one major obstacle in biomedical research is the necessity to validate them on a large collection of tissue specimens. Traditional histopathological techniques are often unacceptably time consuming, extremely labor intensive and very expensive [27]. These limitations have delayed the introduction of novel markers into everyday clinical practice. TMA offers a valuable solution to overcome some of these problems. Using TMA technology many tissue specimens (in our institute, up to 1000 histology blocks) can be arrayed at the same time [28]. Therefore, TMAs are often used for the characterization of antibodies and for tissue specific expression profiling of proteins and genes via *in situ* hybridization [25,28]. Of note, the vast majority of TMAs are analyzed using immunohistochemistry, while a small fraction are investigated by *in situ* hybridisation techniques, such as interphase FISH. Immunohistochemcal analysis is often criticized due to the subjective and semiquantitative means of determining the level of protein expression hampered by the intra laboratory differences in staining procedures, as well as the inter-observer variability. Importantly, compared to the broadly available standard histopathological approach, the TMA methodology allows to analyze all the tissue specimens arrayed in the same manner, for example exposing them to the identical antigen retrieval procedure, reagent concentrations, incubation times with antibodies/probes, thus consequently resulting in an high level of standardization [28]. Thus, the use of TMA has substantial benefits over standard techniques [25]. For example, Diaz and colleagues [29] showed that the evaluation of HER-2 using TMA-IHC survey was correctly scored in over 90% of the tested laboratories. These results further underline that the use of TMAs can reduce variability during the evaluation. Furthermore, among the other major advantages of using TMA, only few quantities of reagents and substantially less laboratory work is required to perform the experiments, making TMA approach exceptionally cost-effective [28]. Additional benefits of using TMA include marginal exhaustion of donor tissue blocks (obviously considered as vital resources) and the possibility to include internal positive and negative controls (cell line materials or tissues with a known expression) while constructing the TMA [30,31]. All together TMA methodology represents a significant advancement in molecular pathology over traditional methods.

## 3. TMA in Hepatocarcinogenesis Research

Tissue specimens represent a fundamental tool for biomedical research. Their analysis is often a crucial step towards the understanding of the molecular background of a disease. For research purposes of liver tumors, TMA technology has been proven to be a reliable and effective tool. However, it is also important to mention that the TMA technology harbors a number of limitations. One potential limitation of using TMA is that the small cores sampled may not be representative of whole tumors [24]. This issue is very critical in particularly heterogeneous cancers such as HCC. For other tumor entities, several studies have addressed this point by comparing TMA analysis results with whole mount sections data. High levels of correlation have been described comparing these procedures in a range of tumor types such as breast, prostate, bladder and human fibroblastic tumors [27,30]. For example, Kononen *et al.* [32] using breast-TMA found the same frequencies of HER-2, c-myc, cyclinD1 and 17q23 amplifications in breast cancer as were expected from previous published literature using whole tissue sections [33]. Interestingly, some studies demonstrated that increasing the number of cores, to compensate for heterogeneity, only slightly increased the rate of data validity [24,28,33]. Conversely, such an approach has the disadvantage of generating significant additional labor work during the arrays preparation [27]. Importantly, it should be mentioned that TMA are intended to estimate the prevalence of a selected markers within a large population of samples, rather then to provide a detailed analysis at the level of single specimen. 

**Figure 1:**
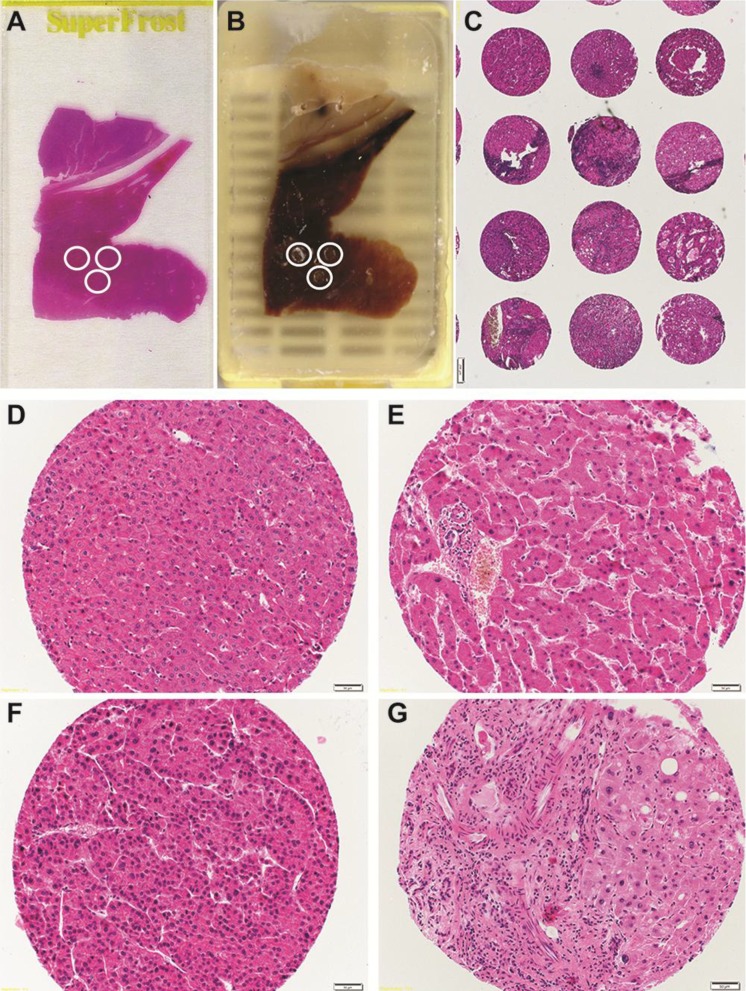
Liver TMA construction steps and examples of different quality cores. (**A**) A HE-stained whole section is evaluated and specific areas are selected. (**B**) Matching tissue areas are punched on the corresponding FFPE block. (**C**) Representative picture of multiple arrayed cores. (**D**) Representative picture of a low quality normal liver sample containing only hepatocyte cells. (**E**) Example of high quality normal liver punch with portal tract and hepatocytes. (**F**) Low quality HCC sample with no other cells than transformed hepatocytes. (**G**) High quality HCC sample containing aportion of normal tissue, portal tract and an HCC area.

For liver focused studies, in case of non-neoplastic liver specimens, it is ideal to evaluate the staining patters of a determined marker over the structure of an entire acinus. It is important to define staining in as many as possible anatomical regions (Figure 1). Thus, it would be optimal to select tissue punches that include at least one portal tract and one central vein. It is conceivable that choosing the 1 mm diameter (or higher) punch is the best option to be selected while constructing a liver TMA. In order to facilitate the cutting and the evaluation procedures, while constructing a TMA it is also important to have convenient spacing between cores of at least 0.15 mm [23]. Furthermore, tissue losses are observed at different percentage depending on the tissue used to construct the TMA [34], with a range from 5% to 33% of used specimens [27], and represent a frequent problem also in liver TMA. The choice of larger tissue punches (e.g., 1 mm compared to 0.6 mm) reduces the frequency of tissue loss [35].

The value of TMAs in the study of liver hepatocarcinogenesis has been proven by a number of studies that employed this technology to unravel some of the key players involved in HCC’s biology. For example, the association of Clusterin, a highly conserved glycoprotein with previously reported pro-tumorigenic function, with metastasis in HCC has been proved by Lau *et al.* [36] using a TMA containing 104 pairs of primary HCCs and their matched metastasis. Clusterin is overexpressed in HCC metastasis and facilities them via modulating YKL-40, a mediator of matrix remodeling processing [37]. Similarly, Hu *et al.* [38], using a TMA composed of 60 pairs of primary/metastatic HCCs, demonstrated that the transcriptional repressor ZHX2 (Zinc-fingers and homeoboxes-2) is altered in HCC and its levels correlate with disease stage. Furthermore, ZHX2 is highly overexpressed in metastatic prone lesions. Additional work performed using a TMA containing primary and recurrent HCCs showed that FGF3 (fibroblast growth factor 3) is associated with HCC recurrence and metastasis [39]. The analysis of liver-TMA with different characteristics, such has the one used by Chen *et al.* [40], generated using HCC and adjacent tissue plus cirrhotic and normal liver specimens, has shown that Heparanase overexpression is linked to HCC prognosis and grade. The expression of KisSS-1, a multi-protein producing genes involved in gonadotropin-releasing hormone and a putative metastasis suppressor in melanoma, was investigated in intra-hepatic HCC metastasis and reported to be lost in these lesions [41]. An extensive survey of putative hepatic stem/progenitor cell biomarkers, namely CK19 (cytokeratin 19), CD133, Nestin and CD44 conducted by Yang and collogues [42] using HCC-TMA revealed that high HSC/HPC profile along with high VEGFA (vascular endothelial growth factor A) levels and increased MVD (micro vascular density) had significant lower OS (overall survival) and RFS (recurrence free survival). More recently, CK19 levels together with CK7 (cytokeratin 7) have been investigated again using an HCC-TMA and found to be significantly associated with tumor grade and AFP levels (alpha-fetoprotein) [43]. Importantly, this work also shows that CK19 expression is extremely rare in pre-cancerous DNs (dysplastic nodules) while increases in small HCCs, suggest CK19 association with disease progression. Conversely, CK7 is already expressed in DNs and further augmented in HCC lesions. The Wnt/β-catenin pathway is among the best-studied and mostly altered one in HCC [11]. Using a liver TMA generated from 179 HCC and matched non-tumorous liver blocks, Cheng *et al.* [44] reported that expression of a histone-lysine *N*-methyltransferase, EZH2 (Enhancer Of Zeste Homolog 2), is significantl associated both with the nuclear and cytoplasmic β-catenin expression. Furthermore, they demonstrated that poorly differentiated HCC presents stronger EZH2 and β-catenin staining compared to both moderate and well-differentiated HCC. In contrast, neither EZH2 nor β-catenin nuclear staining is found in the surrounding liver tissue or in the normal liver specimens, suggesting the importance of Wnt/β-catenin and EZH2 in HCC biology. The use of liver specific TMAs has been reported to be a valuable tool also for the study of the endothelial compartment of the liver. Geraud and colleagues [45] could show that liver sinusoidal endothelial cells (LSEC) undergo a phenotypic switch upon development of chronic liver disease. Specifically, they observed that the typical sinusoidal cell’s fenestrae are lost and a basal membrane is formed, thus leading to the capillarization of liver sinusoids. Taking advantage of our large collection of HCC tissues, our group also contributed to liver carcinogenesis research by reporting that GPC3 (Gypican 3), a heparin sulfate proteoglycan protein with cell proliferation and apoptosis regulatory activity, could be a useful diagnostic marker to differentiate between HCC, non-neoplastic liver disease and pre-neoplastic lesions [46]. In this study, using a multi-tumor TMA, we investigated a total of 4387 tissue samples from 139 tumor types. Our data revealed that GPC3 is expressed in more than about 60% of the investigated HCC specimens, while it is observed in less than 10% of the non-neoplastic liver tissue and in about 16% of pre-neoplastic lesions [46]. Hep Par 1 (Hepatocyte paraffin 1) was proposed as valuable marker to differentiate HCC from other lesions metastasizing to the liver [47]. Using a TMA comprising 3940 tissue samples, we observed that Hep Par 1 is frequently expressed (*ca.* 73%) of analyzed HCC, while in non-hepatic tumors such as lung, gallbladder, pancreas, stomach, small intestine, adenoma of the colon with high-grade dysplasia, adrenal gland carcinoma, paraganglioma and malignant melanoma it is almost virtually absent. Our work suggests that Hep Par 1 is a highly specific marker for HCC [48].

More recently, by analyzing a TMA composed of 69 normal liver specimens, 93 cirrhotic samples and 174 HCCs, we also showed that the SH2D4A (SH2 domain containing 4A) gene is frequently down regulated in HCC, further corroborating its presumptive role as tumor suppressor gene. Finally, for HCC, the use of TMA has revealed a great potential in a comparative study analyzing Asian and American cohort of patients, underling different expression profiles of p53 and MDM2 in the different populations. This approach could represent a novel strategy to identify novel molecular targets based on patient ethnicity [49]. 

## 4. Future Prospective

The vast majority of TMAs so far employed for research purposes in liver tumor studies, have been generated starting from formalin-fixed paraffin-embedded (FFPE) embedded material. More recently fresh frozen tissue-TMAs of cores embedded in optimal cutting temperature (OCT) blocks have been described [30]. TMAs are constructed from unfixed fresh-frozen tissue that has been embedded in a recipient block of OCT media. Such types of TMA present a number of advantages; for example contrary to FFPE-generated TMA where fixatives in the embedded tissue might severely affect the quality of RNA, giving sub-optimal results for RNA hybridization, frozen TMA provide high quality material for study of RNA, DNA and proteins. Indeed, as frozen TMAs are generated from unfixed tissue with antigen preserved structures, these TMAs are extremely useful when antibodies do not work on FFPE tissue, or when FISH-based analysis is required. FISH on TMAs has been frequently used to validate findings of gene amplifications discovered by genome-wide screening [30]. Conversely, one major drawback is that the brittleness of frozen OCT makes coring procedures much more difficult and only fewer samples can be arrayed to avoid cracking of the blocks. In addition, cell morphology in frozen TMAs is of lower quality than in the FFPE counterpart [50]. 

Moreover, in multi-step diseases such as HCC it is of fundamental help to assess molecular changes through the different stages of tumor progression [30]. Generating progressing TMAs can face this issue [28,51]. Progressing TMAs are defined as TMAs containing from normal to hyperplastic, dysplastic lesions up to HCC specimens. Such TMAs have already proven their ability to uncover stage-specific molecular alterations, for example in prostate cancer progression, where the amplification of the Androgen Receptor (AR) gene [52] or the amplification of IGFBP2 locus (insulin-like growth factor binding protein 2) [53] were usually found in hormone refractory end-stage prostate cancers but rarely observed in untreated primary tumors. The assembly of such TMA will be of fundamental help for the study of liver carcinogenesis.

## 5. Conclusions

The rapid and effective translation of molecular based discoveries into new therapeutic targets, useful markers to predict response to therapy, or to help diagnostic assessment is a fundamental issue in modern biomedical research. The TMA approach has proven to have valuable advantages in comparison to standard whole section analysis, as hundreds of tissue samples can be examined in a single experiment. Furthermore, the TMA method provides a judicious use of precious tissue and offers high experimental uniformity. In the current world of high-throughput technology, TMA has revolutionized the histopathological analysis and will be of great help to further advance the knowledge in the field of hepatocarcinogenesis research. 

## Abbreviation

Tissue microarray (TMA), Hepatocellular carcinoma (HCC), hepatitis B virus (HBV), hepatitis C virus (HCV), Immunohistochemistry (IHC).
